# Efficacy of first‐line immune checkpoint inhibitors in patients with advanced NSCLC with *KRAS* , *MET* , *FGFR* , *RET* , *BRAF* , and *HER2* alterations

**DOI:** 10.1111/1759-7714.14448

**Published:** 2022-05-02

**Authors:** Yuji Uehara, Kageaki Watanabe, Taiki Hakozaki, Makiko Yomota, Yukio Hosomi

**Affiliations:** ^1^ Department of Thoracic Oncology and Respiratory Medicine, Tokyo Metropolitan Cancer and Infectious Diseases Center Komagome Hospital Bunkyo‐ku Tokyo Japan; ^2^ Department of Precision Cancer Medicine, Center for Innovative Cancer Treatment, Graduate School of Medical and Dental Sciences Tokyo Medical and Dental University Bunkyo‐ku Tokyo Japan; ^3^ Department of Life Science and Medical Bioscience Waseda University Shinjuku Tokyo Japan

**Keywords:** immunotherapy, *KRAS* mutation, lung cancer, *MET* mutation, oncogenic drivers

## Abstract

**Background:**

In patients with non‐small cell lung cancer (NSCLC) harboring driver alterations, the efficacy of immune checkpoint inhibitors (ICIs) remains uncertain. Our study aimed to examine the first‐line ICI efficacy in patients with NSCLC harboring *KRAS*, *MET*, *FGFR*, *RET*, *BRAF*, and *HER2* alterations in a real‐world setting.

**Methods:**

This single‐center, retrospective cohort study included patients with advanced NSCLC harboring *KRAS*, *MET*, *FGFR*, *RET*, *BRAF*, *HER2* alterations or driver‐negative, and were treated with first‐line ICI therapy. Best overall response, progression‐free survival (PFS), and overall survival (OS) were evaluated.

**Results:**

Seventy‐eight patients with NSCLC were included (median age, 72 years): 67% were men, 15% were never‐smokers, and 83% had adenocarcinoma. The driver alterations involved *KRAS* (*n* = 21), *MET* (*n* = 6), *FGFR* (*n* = 3), *RET* (*n* = 2), *BRAF* (*n* = 2), *HER2* (*n* = 1), and driver‐negative (*n* = 43). The partial responses for *KRAS*, *MET*, *FGFR*, *RET*, *BRAF*, *HER2*, and driver‐negative were 57%, 50%, 100%, 50%, 100%, 0%, and 47%, respectively. The median PFS (months) was 16.2 (95% confidence interval [CI]: 6.3– not reached [NR]) for *KRAS*, 2.8 (95% CI: 2.7–NR) for *MET*, 11.7 (95% CI: 5.9–NR) for other alterations (*FGFR*, *RET*, *BRAF*, and *HER2*), and 10.0 (95% CI: 3.7–14.3) for driver‐negative, respectively. The median OS (months) was 31.3 (95% CI: 9.0–NR) for *KRAS*, not reached for *MET*, 23.5 (95% CI: 18.3–NR) for other alterations, and 21.1 (95% CI: 15.2–NR) for driver‐negative, respectively.

**Conclusions:**

The benefit of the first‐line ICI was similar in advanced NSCLC regardless of the driver alterations, except for *MET* alterations.

## Graphical abstract

The benefit of the first‐line ICI was similar in advanced NSCLC regardless of the drivers, except for *MET* drivers. The median PFS according to the driver subgroup was 16.2 months (95% CI: 6.3–NR) for *KRAS* alterations, 2.8 months (95% CI: 2.7–NR) for *MET* alterations, 11.7 months (95% CI: 5.9–NR) for other alterations (*FGFR*, *RET*, *BRAF*, and *HER2*), 10.0 months (95% CI: 3.7–14.3) for driver‐negative.

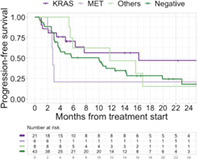

## INTRODUCTION

The management of patients with advanced non‐small cell lung cancer (NSCLC) has significantly improved as a result of the development of targeted therapies and immunotherapy based on molecular testing. The Food and Drug Administration (FDA) has approved inhibitors for the first‐line treatment of NSCLC with *EGFR*, *ALK*, *ROS1*, *RET*, *MET*, *BRAF*, and *NTRK* alterations. For patients with *KRAS* G12C‐mutated NSCLC, sotorasib was approved by the FDA in 2021. For patients with *HER2* alterations, some phase 2 studies with poziotinib or trastuzumab–teruxtecan have shown a high response rate and durable activity.^1^, ^2^ In patients with NSCLC harboring *FGFR* alterations, clinical studies of selective FGFR inhibitors are ongoing.^3^, ^4^ However, most previous clinical trials for targeted therapies in the first‐line setting are single arm studies or compared with chemotherapy without immune checkpoint inhibitors (ICIs), and thus have not been compared with ICI regimens.

In previous clinical trials, the clinical benefit of ICIs for patients harboring *EGFR* and *ALK* alterations was limited.^5^, ^6^, ^7^ Although a meta‐analysis of randomized controlled trials showed that *KRAS* alteration was a positive predictive factor for ICI treatment, multiple retrospective studies have demonstrated conflicting results because of heterogeneous populations.^8^, ^9^, ^10^, ^11^ Moreover, most studies of ICIs have overlooked the other oncogenic drivers (*MET*, *FGFR*, *RET*, *BRAF*, *HER2*, and *ROS1*), and, as a result, the efficacy of ICI remains unclear in patients harboring *KRAS* and other rare oncogenic drivers. Few retrospective studies have evaluated the efficacy of ICI in patients with these rare oncogenic drivers.^12^, ^13^, ^14^, ^15^ Although they have reported mixed results, most of them have shown similar efficacy as in unselected patients with NSCLC. However, these studies with small cohorts were limited to analysis with ICI monotherapy or any line of treatment. The number of prior lines of therapy may lead to varying clinical outcomes.

Herein, we present the efficacy of first‐line ICI in patients with NSCLC against *KRAS*, *MET*, *FGFR*, *RET*, *BRAF*, and *HER2* driver alterations confirmed by next‐generation sequencing (NGS) in a real‐world setting.

## METHODS

### Study design

This retrospective, single‐center, observational study was conducted at the Komagome Hospital in Japan. The study was approved by the Institutional Review Board of the Komagome Hospital (No.2866). The primary objective was to assess ICI efficacy (best overall response, progression‐free survival [PFS], and overall survival [OS]) for NSCLC with *KRAS*, *MET, FGFR*, *RET*, *BRAF*, or *HER2* driver alterations, or driver‐negative, stratified according to the number of patients with driver alterations (*KRAS*, *MET*, Others [*FGFR*, *RET*, *BRAF*, and *HER2*], and driver‐negative).

### Patients

Between May 2019 and July 2021, adult patients with advanced NSCLC were selected if they had confirmed NSCLC harboring *KRAS*, *MET, FGFR*, *RET*, *BRAF*, or *HER2* alterations or driver‐negative, and were treated with first‐line ICI. Patients who were enrolled in a clinical trial of immunotherapy were excluded. We reviewed the medical records and extracted the following patient characteristics: age, sex, smoking status, Eastern Cooperative Oncology Group Performance Status (ECOG‐PS), histology, details of ICI regimens, molecular alterations status, programmed death‐ligand 1 (PD‐L1) status, and survival.

### Molecular diagnostics

Driver alterations of *KRAS*, *MET, FGFR*, *RET*, *BRAF*, or *HER2*, or driver‐negative were identified by NGS of the tumor using the Oncomine Dx Target Test (Ion Torrent PGM Dx Sequencer, Thermo Fisher Scientific), which has been approved by the FDA and Japan's Ministry of Health, Labor and Welfare. This is a hot‐spot panel test using the amplicon method, which analyzes alterations in 46 genes and fusion in 21 genes using DNA and RNA isolated from formalin‐fixed paraffin‐embedded specimens. For *MET* alterations, only exon14 skipping mutations were considered. PD‐L1 expression was evaluated using the PD‐L1 22C3 pharmDx (Dako).

### Statistical analysis

Summary statistics were used to describe the patient characteristics. The best response to ICI treatment was determined through Response Evaluation Criteria in Solid Tumors version 1.1 criteria (RECIST 1.1). PFS was defined as the time from starting treatment until disease progression or death. Progression was defined according to radiological or clinical progression (deteriorated clinical status preventing systemic treatment) or death. OS was defined as the time from starting treatment until death. Patients with continuing therapy without progression at the last follow‐up date were censored for PFS at that date. Patients who were alive at the last follow‐up were censored for OS. PFS and OS data were analyzed using Kaplan–Meier estimation, and the survival endpoints were compared using log‐rank tests. The hazard ratio was calculated by log‐rank test and Cox regression analysis. All *p*‐values <0.05 were considered statistically significant. All statistical analyses were conducted using R version 4.1.1.

## RESULTS

### Patient characteristics (*n* = 78)

**FIGURE 1:**
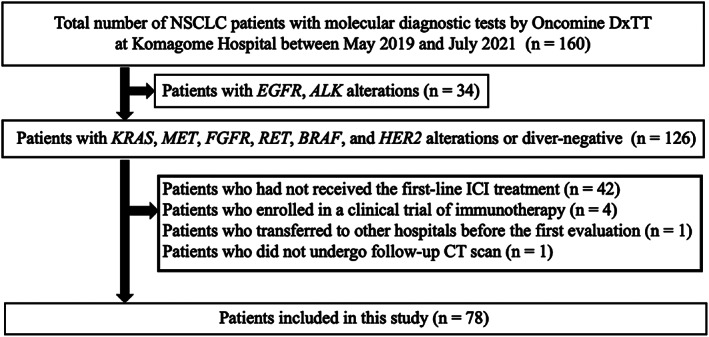
Patient flow of 160 with molecular diagnostic tests by the Oncomine dx target test at Komagome hospital. Abbreviation: ICI, immune checkpoint inhibitors

In this study, the 160 patients with NSCLC with molecular diagnostic tests by Oncomine Dx TT at the Komagome Hospital between May 2019 and July 2021 were identified as candidates for inclusion; among whom, 126 harbored of *KRAS*, *MET, FGFR*, *RET*, *BRAF*, or *HER2* alterations or driver‐negative (Figure 1). We excluded 48 patients for the following reasons: had not received the first‐line ICI treatment (*n* = 42), were enrolled in a clinical trial (*n* = 4), transferred to other hospitals before the first evaluation (*n* = 1), and did not undergo follow‐up computed tomography scan (*n* = 1). We included the remaining 78 patients in the current analysis. The driver alterations involved *KRAS* (*n* = 21), *MET* (*n* = 6), *FGFR* (*n* = 3), *RET* (*n* = 2), *BRAF* (*n* = 2), and *HER2* (*n* = 1), or driver‐negative (*n* = 43) (Table 1). The median age of patients was 72 years (31–89), 52 patients (67%) were men, 12 patients (15%) were never‐smokers, and 68 patients (87%) had a PS of 0–1. Histological assessment showed adenocarcinoma in 65 patients (83%), squamous carcinoma in eight patients (10%), and other carcinoma types in five patients (6.4%). All patients received first‐line ICIs: Pembrolizumab monotherapy for 24 patients (31%), ICI plus chemotherapy for 46 patients (59%), nivolumab and ipilimumab for three patients (3.8%), and nivolumab and ipilimumab plus chemotherapy for five patients (6.4%). PD‐L1 status was available for 68 patients (87%). Positive PD‐L1 expression, defined as tumor proportion score (TPS) of ≥1%, was found in 50 patients (64%), with a high PD‐L1 TPS ≥50% identified in 31 patients (40%).

**TABLE 1:** Patient characteristics (*n* = 78)

	Total (*n* = 78)	KRAS^a^ (*n* = 21)	MET^b^ (*n* = 6)	FGFR^c^ (*n* = 3)	RET^d^ (*n* = 2)	BRAF^e^ (*n* = 2)	HER2^f^ (*n* = 1)	Negative (*n* = 43)
Age at diagnosis, year (range)	72 (31–89)	72 (31–89)	75 (72–86)	55 (46–76)	61 (55–66)	67 (57–77)	54 (54)	68 (42–86)
Sex								
Male	52 (67%)	14 (67%)	3 (50%)	3 (100%)	1 (50%)	0	1 (100%)	30 (70%)
Female	26 (33%)	7 (33%)	3 (50%)	0	1 (50%)	2 (100%)	0	13 (30%)
Smoking								
Never smoker	12 (15%)	2 (10%)	3 (50%)	0	1 (50%)	0	1 (100%)	5 (12%)
Smoker	66 (85%)	19 (90%)	3 (50%)	3 (100%)	1 (50%)	2 (100%)	0	38 (88%)
Performance status								
0–1	68 (87%)	17 (81%)	5 (83%)	3 (100%)	2 (100%)	1 (50%)	1 (100%)	39 (91%)
2	10 (13%)	4 (19%)	1 (17%)	0	0	1 (50%)	0	4 (9.3%)
Histological type								
Adenocarcinoma	65 (83%)	21 (100%)	5 (83%)	1 (33%)	2 (100%)	2 (100%)	1 (100%)	33 (77%)
Squamous	8 (10%)	0	0	2 (67%)	0	0	0	6 (14%)
Other	5 (6.4%)	0	1 (17%)	0	0	0	0	4 (9.3%)
Immunotherapy								
Pembrolizumab	24 (31%)	8 (38%)	4 (67%)	1 (33%)	0	0	0	11 (26%)
ICI + Chemo	46 (59%)	10 (48%)	1 (17%)	2 (67%)	2 (100%)	2 (100%)	0	29 (67%)
Nivolumab + ipilimumab	3 (3.8%)	1 (4.7%)	1 (17%)	0	0	0	0	1 (2.3%)
Nivolumab + ipilimumab + chemo	5 (6.4%)	2 (10%)	0	0	0	0	1 (100%)	2 (4.7%)
PD‐L1 satus								
≧50%	31 (40%)	8 (38%)	4 (67%)	2 (67%)	1 (50%)	1 (50%)	0	15 (35%)
1%–49%	19 (24%)	6 (29%)	0	1 (33%)	0	1 (50%)	1 (100%)	10 (23%)
0%	18 (23%)	3 (14%)	1 (17%)	0	0	0	0	14 (33%)
Unknown	10 (13%)	4 (19%)	1 (17%)	0	1 (50%)	0	0	4 (9.3%)

Abbreviations: Chemo, chemotherapy; ICI, immune checkpoint inhibitors; PD‐L1, programmed death‐ligand 1.

^a^ *KRAS* G12C (*n* = 7), *KRAS* G12D (*n* = 5), *KRAS* G12V (*n* = 4), *KRAS* Q61H (*n* = 3), *KRAS* G12S (*n* = 1), *KRAS* G13C (*n* = 1).

^b^ *MET* exon 14 skipping mutation (*n* = 6).

^c^ *FGFR1* amplification (*n* = 2), *FGFR3* fusion (*n* = 1).

^d^ *RET* fusion (*n* = 2).

^e^ *BRAF* V600E (*n* = 1), *BRAF* D549G (*n* = 1).

^f^ *HER2* G776delinsVC (*n* = 1).

### Best overall response (*n* = 78)

**FIGURE 2:**
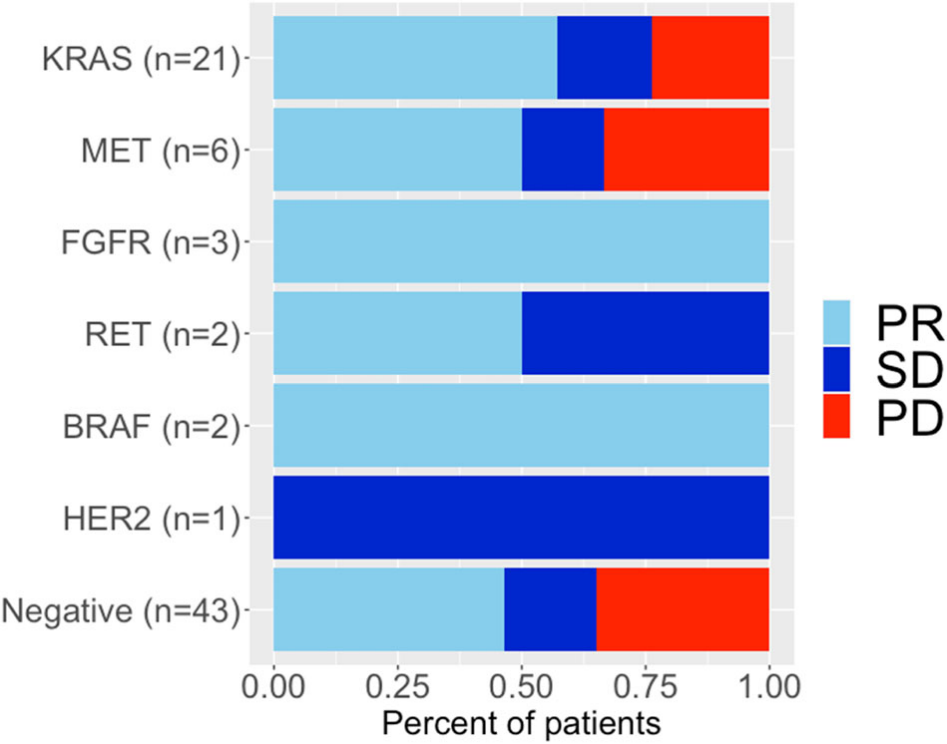
Best response to ICI according to RECIST criteria (*n* = 78). Partial responses were observed in 57% (12/21) patients for *KRAS*, 50% (3/6) for *MET*, 100% (3/3) for *FGFR*, 50% (1/2) for *RET*, 100% (2/2) for *BRAF*, or 0% (0/1) for *HER2* alterations, or 47% (20/43) for driver‐negative. Abbreviations: ICI, immune checkpoint inhibitors; PD, progressive disease; PR, partial response; RECIST, Response Evaluation Criteria in Solid Tumors; SD, stable disease

Among 78 patients with evaluable disease, an objective response rate (ORR) was observed in 41 patients (53%), stable disease (SD) in 15 patients (19%), and progressive disease (PD) in 22 patients (28%). The partial response rates according to the type of driver alteration were 57% (12/21) for *KRAS*, 50% (3/6) for *MET*, 100% (3/3) for *FGFR*, 50% (1/2) for *RET*, 100% (2/2) for *BRAF*, or 0% (0/1) for HER2 alterations, or 47% (20/43) for driver‐negative (Figure 2). Combined analysis of 35 patients with driver alterations (*KRAS*, *MET, FGFR*, *RET*, *BRAF*, and *HER2*) showed that ORR was observed in 21 patients (60%), SD in seven patients (20%), and PD in seven patients (20%). There was no significant difference in ORR between driver alterations and driver‐negative groups (60% vs. 47%, *p* = 0.26).

### Progression‐free survival and overall survival (*n* = 78)

**FIGURE 3:**
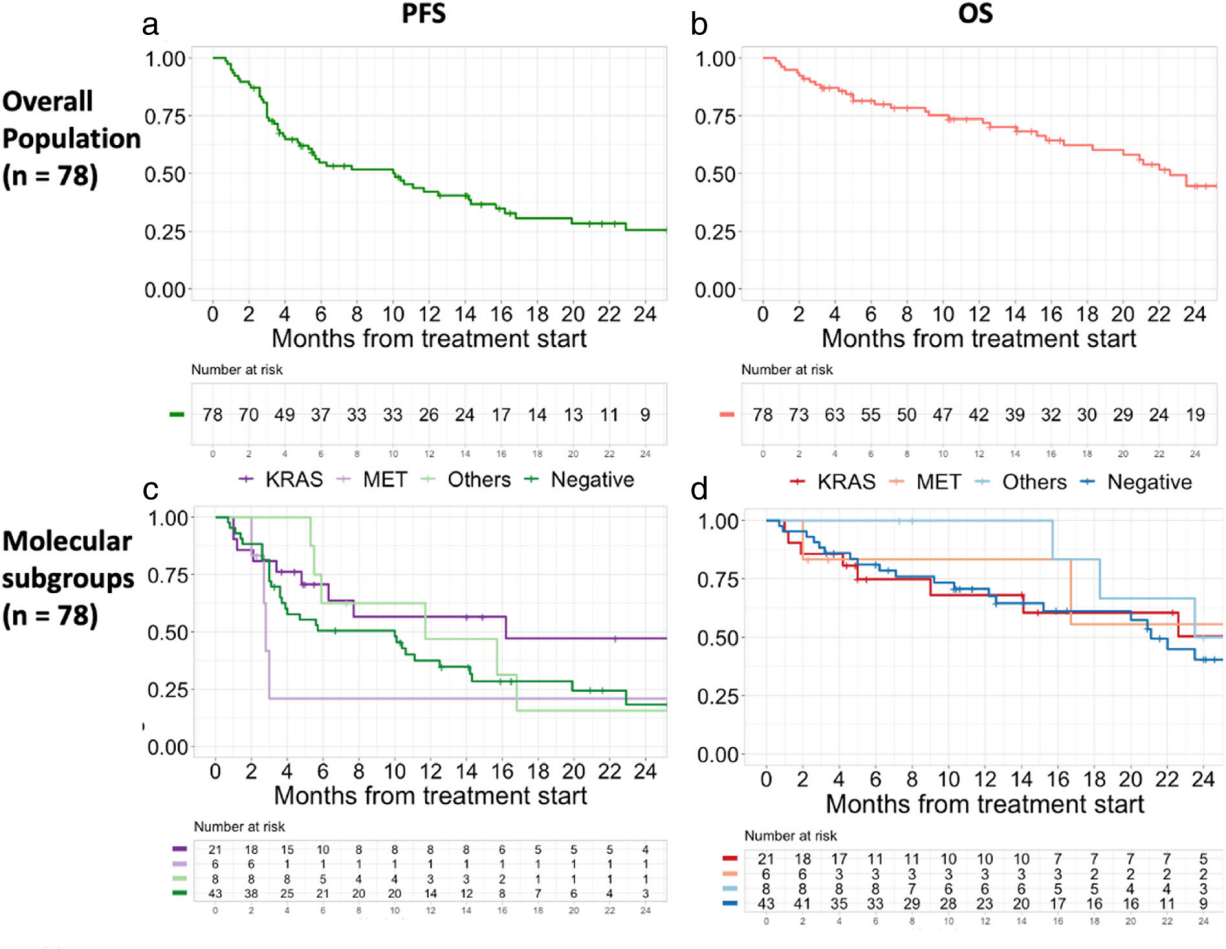
Kaplan–Meier curve for progression‐free survival (PFS) and overall survival (OS) for the entire cohort (a, b) and according to the type of driver alterations (c, d) (*n* = 78). The median PFS for the entire cohort was 10.1 months (95% CI: 5.5–15.7). (a) The median OS for the entire cohort was 22.6 months (95% CI: 18.3 months–NR). (b) The median PFS according to the driver subgroup was 16.2 months (95% CI: 6.3–NR) for *KRAS* alterations, 2.8 months (95% CI: 2.7–NR) for *MET* alterations, 11.7 months (95% CI: 5.9–NR) for other alterations (*FGFR*, *RET*, *BRAF*, and *HER2*), 10.0 months (95% CI: 3.7–14.3) for driver‐negative. (c) The median OS according to the driver subgroup was 31.3 months (95% CI: 9.0–NR) for *KRAS* alterations, not reached for *MET* alterations, 23.5 months (95% CI: 18.3–NR) for other alterations (*FGFR*, *RET*, *BRAF*, and *HER2*), 21.1 months (95% CI: 15.2–NR) for driver‐negative. Abbreviations: CI, confidence interval; NR, not reached; OS, overall survival; PFS, progression‐free survival

**FIGURE 4:**
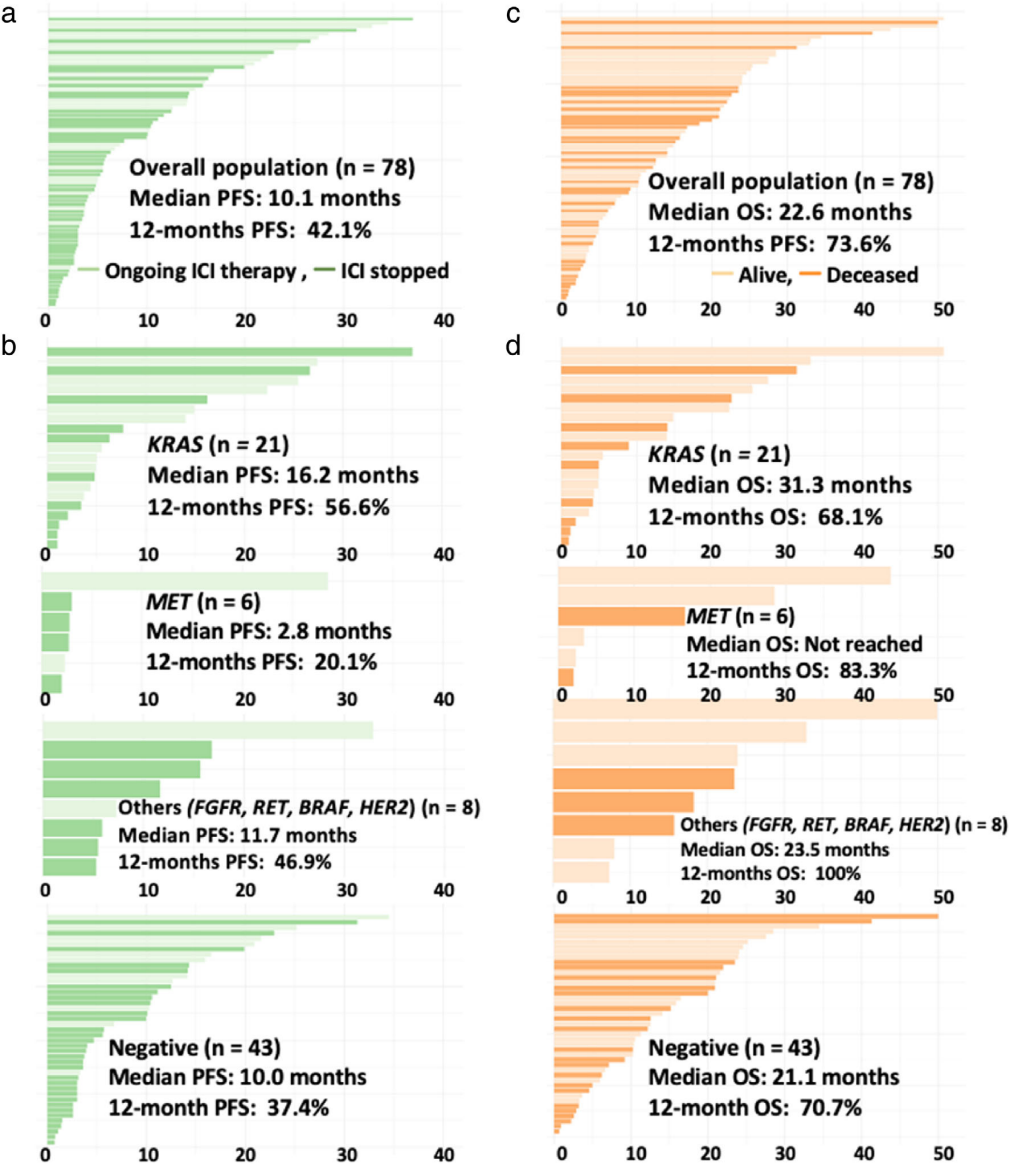
Progression‐free survival (PFS) for the entire cohort (a) and according to the type of driver alterations (b) overall survival (OS) for the entire cohort (c) and according to the type of driver alterations (d) (*n* = 78). The 12‐month PFS rate was 42.1% (95% CI: 31.9%–55.5%) for the overall population, 56.6% (95% CI: 37.3%–85.9%) for *KRAS* alterations, 20.1% (95% CI: 36.8%–100%) for *MET* alterations, 46.9% (95% CI: 21.5%–100%) for other alterations (*FGFR*, *RET*, *BRAF*, and *HER2*), and 37.4% (95% CI: 25.1%–55.8%) for driver‐negative. The 12‐month OS rate was 73.6% (95% CI: 64.0%–84.7%) for the overall population, 68.1% (95% CI: 49.6%–93.5%) for *KRAS* alterations, 83.3% (58.3%–100%) for *MET* alterations, 100% (95% CI: 100%–100%) for other alterations (*FGFR*, *RET*, *BRAF*, and *HER2*), and 70.7% (95% CI: 58.0%–86.2%) for driver‐negative. Abbreviations: CI, confidence interval; OS, overall survival

The median follow‐up period for the censored cases was 13.3 months. The median PFS for the entire cohort was 10.1 (95% confidence interval [CI]: 5.5–15.7) months, and the 12‐month PFS rate was 42.1% (95% CI: 31.9%–55.5%) (Figures 3 and 4). The median PFS (in months) for individual driver subgroups was 16.2 (95% CI: 6.3– not reached [NR]) for *KRAS*, 2.8 (95% CI: 2.7–NR) for *MET*, 11.7 (95% CI: 5.9–NR) for other alterations (*FGFR*, *RET*, *BRAF*, and *HER2*), and 10.0 (95% CI: 3.7–14.3) for driver‐negative. Combined analysis of 35 patients with driver alterations (*KRAS*, *MET*, *FGFR*, *RET*, *BRAF*, and *HER2*) showed that the median PFS was 11.7 months (95% CI: 5.5–NR), and the driver alterations (driver‐positive vs. driver‐negative) were not significantly associated with the median PFS (11.7 vs. 10.0 months, *p* = 0.23).

The median OS for the entire cohort was 22.6 (95% CI: 18.3–NR) months, and the 12‐month OS rate was 73.6% (95% CI: 64.0%–84.7%) (Figures 3 and 4). The median OS (in months) for individual driver subgroups was 31.3 (95% CI: 9.0–NR) for *KRAS*, not reached for *MET*, 23.5 (95% CI: 18.3–NR) for others alterations (*FGFR*, *RET*, *BRAF*, and *HER2*), and 21.1 (95% CI: 15.2–NR) for driver‐negative. Combined analysis of 35 patients with driver alterations (*KRAS*, *MET*, *FGFR*, *RET*, *BRAF*, and *HER2*) showed that the median OS was 23.5 (95% CI: 16.7–NR), and the driver alterations (driver‐positive vs. driver‐negative) were not significantly associated with the median OS (23.5 vs. 23.1 months, *p* = 0.35).

### *KRAS* mutation subgroup analysis (*n* = 21)

**FIGURE 5:**
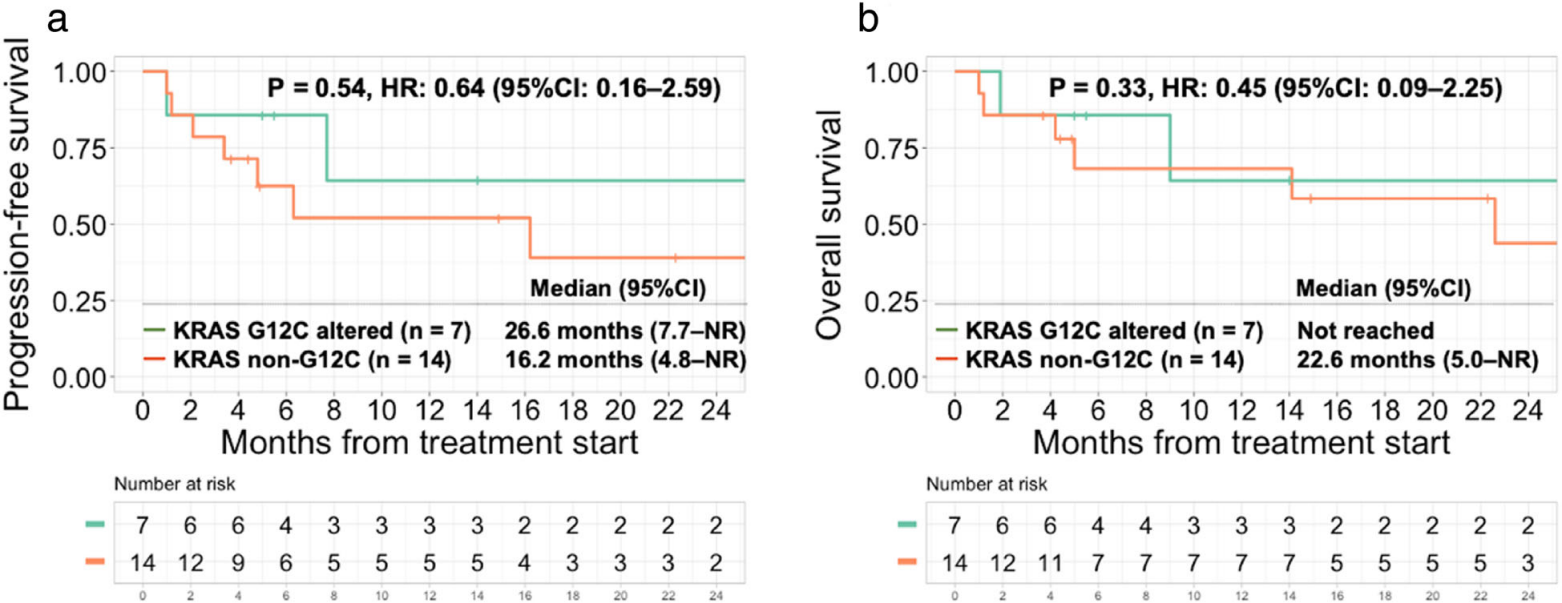
Kaplan–Meier curve for progression‐free survival (PFS) and overall survival (OS) based on *KRAS* mutation subtypes (a, b) (*n* = 21). (a) Compared to *KRAS* non‐G12C mutations (*n* = 14), *KRAS* G12C mutation cases (*n* = 7) showed no significant difference in PFS (HR, 0.64; 95% CI: 0.16–2.59; *p* = 0.54). (b) Compared to *KRAS* non‐G12C mutations (*n* = 14), *KRAS* G12C mutation cases (*n* = 7) showed no significant difference in PFS (HR, 0.45; 95% CI: 0.09–2.25; *p* = 0.33). Abbreviations: CI, confidence interval; HR, hazard ratio; OS, overall survival; PFS, progression‐free survival

Comparing the *KRAS* G12C mutations (*n* = 7) to the *KRAS* non‐G12C mutations (*n* = 14), there was no significant difference in the ORR (71% vs. 50%, *p* = 0.64). *KRAS* mutation status (*KRAS* G12C vs. other *KRAS* mutations) was not significantly associated with median PFS (26.6 vs. 16.2 months, *p* = 0.54) or median OS (NR vs. 22.6 months, *p* = 0.33) (Figure 5).

## DISCUSSION

This retrospective study evaluated patients with NSCLC harboring *KRAS*, *MET*, *FGFR*, *RET*, *BRAF*, and *HER2* driver alterations treated with first‐line ICIs. In patients with driver alterations (*KRAS*, *FGFR*, *RET*, *BRAF*, and *HER2*), the median PFS was similar to that in driver‐negative patients; however, patients with *MET* alterations showed a shorter median PFS of 2.8 months.

While a few retrospective studies have evaluated the efficacy of ICI in patients with rare oncogenic drivers (*MET*, *RET*, *BRAF*, *HER2*), these studies included patients with any line of treatment or patients who only received ICI monotherapy (anti‐PD‐1/PD‐L1antibody) or dual ICI therapy (anti‐PD‐1 and anti‐CTLA‐4 antibodies).^12^, ^13^, ^14^, ^15^, ^16^, ^17^, ^18^ Although most of the ICI efficacy for NSCLC with driver alterations other than *EGFR* and *ALK* appeared similar to those for driver‐negative patients, previous studies have reported mixed results due to heterogeneous populations. Our study included only patients treated with a first‐line ICI, reducing the heterogeneity introduced by the patients' background.

Our study demonstrated patients with *MET* alterations had a shorter median PFS of 2.8 months, compared to those with other driver alterations and driver‐negative. Consistent with our finding, in previous retrospective studies of patients with *MET* alterations, the outcomes achieved with ICIs were poor: median PFS of 2–5 months.^12^, ^13^, ^14^, ^18^, ^19^ In our cohort, one patient with *MET* alterations presented with pneumonitis as an immune‐related adverse event (irAEs), preventing the use of MET inhibitors. A recent retrospective cohort showed that six of seven patients who received MET inhibitors following ICI presented with an early grade ≥3 AE (4 transaminitis, 2 pneumonitis), leading to the permanent discontinuation of MET inhibitors in 3 of the six patients.^20^

Two MET inhibitors approved by the FDA have shown durable clinical benefits: capmatinib, with a median PFS of 12.4 months (GEOMETRY mono‐1 study), and tepotinib, with a median PFS of 8.5 months (VISION study) in treatment naïve patients.^21^, ^22^ These data suggest that the first‐line ICI treatment should be avoided in patients with *MET* alterations before starting MET inhibitors. Previous studies have shown that PD‐L1 status and higher tumor mutational burden (TMB) were not associated with the response in patients with *MET* alterations.^13^ The reasons for the poor outcomes achieved with immunotherapy in *MET* alterations remain largely unclear.

In our result, the efficacy of ICI was favorable in patients with *KRAS* alterations. Retrospective studies in western populations reported that the clinical benefit of ICIs with *KRAS* alterations was similar or better to those without KRAS alterations.^10^, ^11^, ^23^, ^24^ A post‐hoc analysis of KEYNOTE‐042 (first‐line Pembrolizumab monotherapy vs. chemotherapy in PD‐L1 positive advanced NSCLC) showed that patients with *KRAS* alterations had better PFS compared to those without *KRAS* alterations (median PFS: 12 months [*n* = 30] vs. 6 months [*n* = 127]).^8^

Our cohort with *KRA*S alterations showed a remarkable median PFS of 16.2 months and an OS of 31.3 months compared to those reported in western populations, although we could not conduct a direct comparative analysis.^10^, ^11^, ^23^, ^24^ Little is known about the ICI efficacy among Asian patients with lung cancer harboring *KRAS* alterations owing to the lower prevalence of these alterations compared to that in western populations. Indeed, the incidence of NSCLC with *KRAS* alterations in Asian patients was less than 10%.^25^, ^26^, ^27^ Two retrospective Chinese NSCLC cohorts who received ICI monotherapy (any line of treatment) showed that patients with *KRAS* alterations had a longer PFS than those without *KRAS* alterations (longer than 15 months [*n* = 14] vs. shorter than 5 months [*n* = 30]), as well as a longer OS (33 months [*n* = 12] vs. 22 months [*n* = 48]), consistent with our remarkable outcome.^28^, ^29^ A multicenter Asian cohort of 216 patients with NSCLC harboring *KRAS* alterations (ATORG‐005) showed that the patients treated with ICIs had a longer median OS compared to those without ICIs; however, most of their first‐line treatment was chemotherapy, including only 20 patients who received first‐line ICI therapy.^30^ To the best of our knowledge, no large Asian study has analyzed patients with *KRAS*‐altered NSCLC who received immunotherapy.

Recent studies have reported an association between PD‐L1 status and ICI efficacy in patients with *KRAS* alterations, but no significant association between *KRAS* mutational subtypes and ICI efficacy.^10^, ^18^, ^30^, ^31^ The biology of these patients was categorized into three groups by co‐occurring genetic alterations with different immunogenic profiles and responses to ICI.^32^, ^33^ We confirmed the evident favorable clinical efficacy of the first‐line ICI in these patients (*KRAS* G12C mutations and *KRAS* non‐G12C mutations). Although we could not obtain the coalterations because of the limitation of targeted NGS, our remarkable PFS results with Asian patients might be explained by differences between Asians and Caucasians. Further research is needed to focus on the efficacy of ICI for Asian patients with lung cancer harboring *KRAS* alterations.

Our findings do not support decreased efficacy of the first‐line ICI in patients with driver alterations, except for those with *MET* alterations. However, the small number of patients in our cohort precludes firm conclusions regarding the comparative efficacy of ICI and targeted therapy. Therefore, further investigation is necessary to compare the efficacy and safety of different approaches (first‐line ICI regimens and second‐line targeted therapy vs. first‐line targeted therapy and second‐line ICI regimens vs. first‐line ICI regimens in combination with targeted therapy).

We acknowledge that there are several limitations in this study. First, because of its retrospective nature and the small number of patients, the results are only hypothesis‐generating. Second, the response rates and PFS might be overestimated due to the lack of central radiological evaluation and uniform intervals for tumor response assessment. Third, PD‐L1 status was heterogeneous, and our first‐line ICI regimens were different, including chemotherapy which could confound the clinical outcome. Fourth, the TMB and coalterations could not be obtained. Nevertheless, previous studies also possessed these limitations. Our real‐world analysis of patients who received first‐line ICIs support the further comparative analysis to determine the benefit of the first‐line ICI versus targeted therapy use in patients with rare driver alterations.

In conclusion, the clinical benefit of the first‐line ICI was similar in advanced NSCLC, regardless of the driver alteration (*KRAS*, *FGFR*, *RET*, *BRAF*, and *HER2*). However, the outcome for patients with *MET* alterations was inferior; thus, in these patients, the first‐line ICI may be considered after targeted therapy, given their AEs. Due to the small patient numbers in the current analysis, larger studies are warranted to validate our findings.

## CONFLICT OF INTEREST

Yuji Uehara has no conflict of interest to declare. Kageaki Watanabe has received honoraria for speakers from AstraZeneca, Chugai Pharmaceutical, MSD, Eli Lilly Japan, Boehringer Ingelheim, Takeda Pharmaceutical, Bristol Myers Squibb, Merck Biopharma, and Ono Pharmaceutical outside the submitted work. Taiki Hakozaki has received Payment for speakers' bureaus from Chugai Pharmaceutical outside the submitted work. Makiko Yomota has received honoraria for speakers from Chugai Pharmaceutical, Ono Pharmaceutical, AstraZeneca, TAIHO Pharmaceutical, Takeda Pharmaceutical, Boehringer Ingelheim, and Pfizer, outside the submitted work. Yukio Hosomi has received honoraria for speakers from AstraZeneca, Ono Pharmaceutical, MSD, Taiho Pharmaceutical, Pfizer Takeda Pharmaceutical, and Chugai Pharmaceutical outside the submitted work.
